# Molecular Characterization of Pseudomonas aeruginosa Clinical Isolates Through Whole-Genome Sequencing: A Comprehensive Analysis of Biotypes, Sequence Types, and Antimicrobial and Virulence Genes

**DOI:** 10.7759/cureus.71118

**Published:** 2024-10-09

**Authors:** Vallab Ganesh Bharadwaj, Tarun Kumar Suvvari, Venkataramana Kandi, Chitra Rajalakshmi P, Milankumar V Dharsandia

**Affiliations:** 1 Microbiology, Trichy Sri Ramasamy Memorial Medical College Hospital and Research Centre, Tiruchirapalli, IND; 2 General Medicine, Rangaraya Medical College, Kakinada, IND; 3 Research, Squad Medicine and Research (SMR), Visakhapatnam, IND; 4 Clinical Microbiology, Prathima Institute of Medical Sciences, Karimnagar, IND; 5 Microbiology, Peoples Medical College and Research Centre, Bhopal, IND

**Keywords:** antimicrobial resistance, esbl, genotype, next-generation sequencing (ngs), phenotype, pseudomonas aeruginosa, virulence, whole-genome sequencing

## Abstract

Introduction

*Pseudomonas aeruginosa* (PA) is a bacterial species commonly isolated from human clinical specimens. Despite being present in the environment as a saprophyte, PA possesses the ability to cause human infections, especially among debilitated patients. It is therefore essential to understand the genomic imprints of antimicrobial resistance (AMR) and virulence genes associated with PA isolated from patient samples.

Methods

The study carried out next-generation sequencing (NGS) or whole-genome sequencing (WGS) of nine PA strains isolated from various human clinical specimens from patients at Prathima Institute of Medical Sciences, Karimnagar, India. All the isolates were identified by conventional microbiological methods and confirmed by automated systems. Antimicrobial susceptibility patterns of the isolates were carried out using the Kirby-Bauer disc diffusion method. Additionally, NGS/WGS was done to evaluate the carriage of AMR and virulence genes associated with each PA strain. Sequence type was identified through multi-locus sequence typing (MLST).

Results

The genotype and phenotypic antimicrobial susceptibility patterns revealed the same (11.11% resistance) results with carbapenems and fluoroquinolone antibiotics. However, discordant antimicrobial susceptibility patterns were noticed with trimethoprim-sulfamethoxazole (66.66% resistance phenotype vs. 100% sensitive genotype), aminoglycosides (100% sensitive phenotype vs. 100% resistant genotype), and beta-lactamase/extended-spectrum beta-lactamase (ESBL) (44.44% sensitive phenotype vs. 100% resistant genotype) antibiotics. All (100%, 9/9) the PA isolates included in the study demonstrated the presence of multiple antibiotic resistance and virulence genes. The antibiotic resistance genes identified included *aph*, *aad*, *aac*, *bla*_PDC_, *bla*_OXA_, *bla*_VIM_, *catB7*, *fosA*, *qnrVC1*, and *crpP*. All (100%, 9/9) isolates demonstrated the presence of class C beta-lactamase *bla*_PDC_ and class B metallo-beta-lactamase *bla*_OXA_. Only one (11.11%, 1/9) isolate showed the presence of subclass B1 metallo-beta-lactamase *bla*_VIM_. Among the virulence genes identified were *toxA*, *fih*, *xcp*, *wzz*, *pvc*, *pvd*, and many others. This study showed the presence of ST244, a high-risk PA strain with global significance.

Conclusions

PA is an opportunistic pathogen, and its isolation among hospitalized patients should be carefully evaluated. Tracking PA for the presence of high-risk sequence types and the prevalence of resistance and virulence genes could improve the understanding of the organism. Molecular data obtained from this study demonstrated that the PA isolates carried multiple antibiotic-resistant and virulence genes that could potentially enable them to cause invasive infections and treatment failures. The data obtained from this study could be applied to devise treatment and management strategies favorable to the hospital or healthcare institution.

## Introduction

*Pseudomonas *is a ubiquitous bacterium living in the environment, especially near human habitats [1]. It is a saprophytic bacteria found in water and soil, including hospital environments [2]. However, *Pseudomonas aeruginosa* (PA) has been associated with serious infections in hospitals known as nosocomial infections, hospital-acquired infections, or healthcare-associated infections (HAIs). These infections are generally noted among debilitated patients like newborn babies/neonates, intubated patients, critically ill patients, and patients admitted to intensive care units (ICUs), among others [3-5]. *Pseudomonas *species (spp.) are also known to contaminate antiseptics, survive in the harshest environments, and acquire multi-drug resistance (MDR) [6]. PA is an established opportunistic pathogen that uses the weakened host defense system to cause invasive diseases [7]. The infection of the lungs in patients suffering from cystic fibrosis is one of the best examples of how PA utilizes the compromised host environment and defenses to establish itself to cause disease. PA can survive in biotic and abiotic environments, intrinsically develop antibiotic resistance, form biofilms, and use quorum sensing and other adaptive mechanisms [2].

*Pseudomonas *has been named under the critical group of bacteria against which there is an urgent need to develop newer antimicrobial agents by the World Health Organization (WHO) [8]. Moreover, PA demonstrates MDR is an important member of the ESKAPE (*Enterococcus faecium*, *Staphylococcus aureus*, *Klebsiella pneumoniae*, *Acinetobacter baumannii*, PA, and *Enterobacter*) pathogens [9]. Infections with PA can become difficult to treat due to MDR. PA is known to develop chromosomal mutation-induced antibiotic resistance. Additionally, PA can acquire resistance through gene transfer mechanisms, including horizontal gene transfers (HGTs) and the transfer of mobile genetic elements like plasmids and transposons [10].

Given the ubiquitous nature of PA and its ability to survive in the environment and cause mild to severe infections, especially among hospitalized patients, it is essential to understand the virulence mechanisms of this bacterium. This study was carried out among PA isolated from different human clinical conditions. The genotypic and phenotypic antibiotic susceptibility patterns, AMR and virulence genes, and sequence types were evaluated.

## Materials and methods

An observational, analytical, and cross-sectional study was conducted among patients attending the Prathima Institute of Medical Sciences (PIMS), Karimnagar, India, between January 2019 and January 2022. Nine PA bacterial isolates showing MDR were collected. The samples collected were pus (five), respiratory specimens including sputum (two) and endotracheal secretions (one), and blood (one). The identification of bacteria and antimicrobial susceptibility testing (AST) of isolates was determined using both conventional methods and VITEK® (bioMérieux, Inc., USA), an automated identification system [11,12]. The results were interpreted as per the Clinical and Laboratory Standards Institute (CLSI) guidelines [13].

Bacterial identification

Conventional methods, including cultural characteristics, biochemical reactions, and pigment production, identified PA. The bacteria appearing as gram-negative bacilli on Gram stain, motile, positive for oxidase, catalase, and citrate utilization, and negative results for urease, along with non-lactose fermenting colonies on Mackonkey's agar and greenish diffusible pigment on nutrient agar, were established as PA. All isolates were confirmed using VITEK® (bioMérieux, Inc.).

Antimicrobial susceptibility testing

The Kirby-Bauer disk diffusion method was applied to carry out antibiotic susceptibility testing. Two to three pure PA colonies from overnight bacterial growth (inoculum) were taken from the culture plate. The inoculum was mixed well into the peptone water/sterile saline. Later, it was incubated at 37°C for 1-2 hours. The test tube now shows growth in the form of turbidity. The turbidity was adjusted to match McFarland standards. The McFarland standards were achieved manually by comparing and adjusting the culture turbidity with a solution prepared by mixing 0.05 mL of 1% barium chloride and 9.95 mL of 1% sulfuric acid.

Later, sterile cotton swabs were used to prepare the test organisms' lawn culture/carpet culture on Mueller-Hinton agar (MHA). Different antibiotic-impregnated filter paper disks were applied with the help of sterile forceps. The plates were incubated overnight at 37°C for 12-18 hours. After the incubation, organisms sensitive/susceptible to the antibiotic fail to grow near the antibiotic discs, forming a zone of inhibition/clearance, measured in millimeters (mm). However, bacteria resistant to an antibiotic grow closer/toward the edge of the antibiotic-impregnated disk. PA isolates were designated MDR when they demonstrated resistance to at least one agent in three or more antimicrobial classes.

The antibiotic classes tested were carbapenem group-imipenem (IPM) (10 µg); aminoglycosides-amikacin (AK) (30 µg), gentamicin (GEN) (10 µg); fluoroquinolones-ciprofloxacin (CIP) (5 µg), ofloxacin (OF) (5 µg); sulphonamide-trimethoprim-sulfamethoxazole (TSX) (1.25/23.75 µg); Cephalosporins-ceftazidime (CAZ) (10 µg), ceftriaxone (CTR) (30 µg), cefotaxime (CTX) (30 µg), cephalothin (30 µg); beta-lactamase/extended-spectrum beta-lactamase (ESBL)-piperacillin-tazobactam (PTZ) (30/6 µg). PA ATCC 27853 was used as a control strain.

Whole-genome sequencing (WGS)/next-generation sequencing (NGS)

Genomic deoxyribonucleic acid (DNA) was extracted from PA isolates using the Qiagen QIAamp DNA Mini kit (Qiagen, Hilden, Germany) following the manufacturer’s instructions. Double-stranded DNA libraries with 450 base pairs (bp) insert size were prepared and sequenced on the Illumina platform with 150 bp paired-end chemistry. The genomes that passed sequence quality control were assembled using Spades v3.14 [14] to generate contigs and annotated with Prokka v1.5 [15]. The species identification was done using bactinspector and contamination was assessed using confindr. All the quality metrics were combined using MultiQC and Qualifyr to generate web-based reports. Multi-locus sequence typing (MLST), AMR, and virulence factors were identified using ARIBA tool v2.14.4 with BIGSdb-Pasteur MLST database, National Center for Biotechnological Information (NCBI) AMR acquired gene, and PointFinder databases and VFDB, respectively [16-19]. All the bioinformatic analysis was performed using Nextflow pipelines developed as a part of the Global Health Research Unit (GHRU), United Kingdom, for AMR surveillance as detailed in www.protocols.io.

## Results

Among the samples included, five (55.56%) were pus, four (44.44%) were respiratory specimens, including sputum and endotracheal secretions, and one (11.11%) was blood. The age of the patients included in the study was 43.33±25.96 years, and among them, four (44.44%) were females and five (55.56%) were males. The genotype and phenotypic antimicrobial susceptibility patterns revealed the same (11.11% resistance) results with carbapenems and fluoroquinolone antibiotics. However, discordant patterns were noticed with co-trimoxazole (66.66% resistance phenotype vs. 100% sensitive genotype), aminoglycosides (100% sensitive phenotype vs. 100% resistant genotype), and beta-lactamase/extended-spectrum beta-lactamase (ESBL) (44.44% sensitive phenotype vs. 100% resistant genotype) antibiotics (Table 1).

**Table 1:** Comparison of phenotypic and genotypic antimicrobial susceptibility patterns PAE: *Pseudomonas aeruginosa;* CAR: carbapenems; AGY: aminoglycosides; FLU: fluoroquinolones; BLA/ESBL: beta-lactamase/extended-spectrum beta-lactamase; TSX: trimethoprim-sulfamethoxazole; S: sensitive; R: resistant *: phenotypically sensitive but genotypically resistant; ^#^: phenotypically resistant but genotypically sensitive

Strain	Antibiotic susceptibility patterns									
	Phenotype					Genotype				
	CAR	AGY	FLU	BLA/ESBL	TSX	CAR	AGY	FLU	BLA/ESBL	TSX
PAE-470	S	S*	S	R	R^#^	S	R	S	R	S
PAE-471	S	S*	S	R	R^#^	S	R	S	R	S
PAE-472	S	S*	S	R	R^#^	S	R	S	R	S
PAE-473	R	S*	R	R	S	R	R	R	R	S
PAE-474	S	S*	S	S*	R^#^	S	R	R	R	S
PAE-475	S	S*	S	R	R^#^	S	R	S	R	S
PAE-476	S	S*	S	S*	S	S	R	S	R	S
PAE-477	S	S*	S	S*	R^#^	S	R	S	R	S
PAE-478	S	S*	S	S*	S	S	R	S	R	S

All (100%, 9/9) the PA isolates included in the study demonstrated the presence of multiple antibiotic resistance genes (Table 2).

**Table 2:** Antimicrobial resistance genes identified in this study

Gene	Function
aac(6')-Il	Aminoglycoside N-acetyltransferase
aadA10	ANT(3'')-Ia family aminoglycoside nucleotidyltransferase
aph(3')-IIb	Aminoglycoside O-phosphotransferase
*bla*_OXA_	Oxacillin-hydrolyzing class D beta-lactamase
*bla*_OXA-50_	Oxacillin-hydrolyzing class D beta-lactamase
*bla*_OXA-904_	Oxacillin-hydrolyzing class D beta-lactamase
*bla*_VIM-2_	Subclass B1 metallo-beta-lactamase
*bla*_PDC-1_	Cephalosporin-hydrolyzing class C beta-lactamase
*bla*_PDC-5_	Extended-spectrum class C beta-lactamase
*bla*_PDC-6_	Class C beta-lactamase
*bla*_PDC-8_	Class C beta-lactamase
*bla*_PDC-23_	Class C beta-lactamase
*bla*_PDC-24_	Class C beta-lactamase
*bla*_PDC-41_	Class C beta-lactamase
catB7	Type B-4 chloramphenicol O-acetyltransferase
crpP	Ciprofloxacin resistance protein
fosA	Fosfomycin resistance glutathione transferase
qnrVC1	Quinolone resistance pentapeptide repeat protein

The antibiotic resistance genes identified included aph, aad, aac, *bla*_PDC_, *bla*_OXA_, *bla*_VIM_, *catB7*, *fosA*, *qnrVC1*, and *crpP*. All (100%, 9/9) isolates demonstrated the presence of class C beta-lactamase *bla*_PDC_ and class B metallo-beta-lactamase *bla*_OXA_. Only one (11.11%, 1/9) isolate showed the presence of subclass B1 metallo-beta-lactamase *bla*_VIM_. Further, a strain-wise description of AMR genes is detailed in Table 3.

**Table 3:** Strain-wise description of antimicrobial resistance genes PAE: *Pseudomonas aeruginosa*; M: male; F: female; D: days

Strain	Age/sex	Source of specimen	Clinical diagnosis	Antibiotic resistance genes detected
PAE-470	54/F	Pus	Diabetic foot ulcer	*aph(3')-IIb*, *bla*_PDC-5_, *bla*_OXA-494_, *catB7*
PAE-471	1D/F	Blood	Preterm with respiratory distress	*aph(3')-IIb*, *bla*_PDC-1_, *bla*_OXA_, *fosA*, *catB7*
PAE-472	68/M	Pus	Postoperative femoral graft	*aph(3')-IIb*, *bla*_PDC-23_, *bla*_OXA-904_, *catB7*
PAE-473	12D/F	Endotracheal aspirate	Respiratory distress	*aadA10*, *aac(6')-Il*, *bla*_PDC-41_, *bla*_VIM-2_, *qnrVC1*, *crpP*
PAE-474	54/M	Sputum	Type 2 respiratory failure	*aph(3')-IIb*, *bla*_PDC-6_, *bla*_OXA-50_, *fosA*,* catB7*, *crpP*
PAE-475	67/M	Sputum	Neurosurgery	*aph(3')-IIb*, *bla*_PDC-5_, *bla*_OXA-494_, *catB7*, *fosA*
PAE-476	64/M	Pus	Cellulitis	*aph(3')-IIb*, *bla*_PDC-24_, *bla*_OXA-50_, *fosA*, *catB7*
PAE-477	57/M	Pus	Suture site infection	*aph(3')-IIb*, *bla*_PDC-8_, *bla*_OXA-494_, *catB7*, *fosA*
PAE-478	26/F	Pus	Breast abscess	*aph(3')-IIb*, *bla*_PDC-8_, *bla*_OXA-494_, *catB7*, *fosA*

Among the virulence genes identified were *toxA*, *fih*, *xcp*, *wzz*, *pvc*, *pvd*, and many others. All these genes contribute to bacterial colonization, proliferation, and survival in the host, countering their responses (Table 4).

**Table 4:** Detailed description of the virulence genes identified in this study ATP: adenosine tri-phosphate; RNA: ribonucleic acid; DNA: deoxyribonucleic acid

Gene locus	Name of the gene	Function	Secretory system/source	Effect on the host
*alg44*, 8, *algA*-*G*,* I-L*, *algP*/*algR3*, *Q*-*R*, *U*, *W*-*X*, *Z*	Alginate polysaccharide	Alginate biosynthesis protein	Type III	Biofilm formation
aprA	Alkaline phosphatase, aeruginolysin	Alkaline protease secretion outer membrane protein AprA, alkaline metalloproteinase	Type I	Destroy tissues, basal lamina, hemorrhagic tissue necrosis, inactivate key immune cells,
catB	Muconate cycloisomerase	Muconate cycloisomerase I	Mandelate racemase/muconate lactonizing enzyme family	Isomerase activity, amino acid metabolic process, degradation of aromatic compounds
chpE	Chemotactic transduction protein	Chemotaxis protein, transcriptional regulator	Type III	Chemotaxis
*cheY*, *Z*	Chemotaxis protein	Chemotaxis protein	Type VI	Responsible for chemotaxis, adhesion, motility, biofilm, *CheY* as a tumbling signal, and *CheZ* as a smooth-swimming signal to control flagellar rotation
*clpB*, *clpV1*	*ClpV* is a member of the AAA+ protein family	ATP-binding protease component	Type VI	*ClpB*-thermotolerance, *ClpV1*-ATPase activity, the release of toxins targeting both eukaryotic and prokaryotic species biofilm formation, interbacterial interactions, acute and chronic infections, and stress response
*exoS*,*T*, *Y*	Exoenzyme	Exoenzyme S, exoenzyme T, adenylate cyclase-*exoY*	Type III	Antiphagocytic, cytotoxic activities
*exsA*-*E*	AraC-type transcription activator *ExsA*	exoenzyme S synthesis protein C, transcriptional regulator ExsA, ExsE protein	Type III	Protein cascade results in cytotoxicity and is calcium-dependent
fepD	ferric enterobactin transporter protein	ferric enterobactin transporter protein	Cytoplasmic membrane protein	Import iron into the cell
*hcp1*, *hcpA*	Hemolysin co-regulated protein	Extracellular, secreted protein Hcp, secreted protein HcpA	Type VI	*Hcp1*-heme carrier influences body iron stores
*icmF1*/*tssM1*	Insulin-cleaving metalloproteinase	Insulin-cleaving metalloproteinase outer membrane protein-*icmP*	Type VI	tssM releases toxins targeting both eukaryotic and prokaryotic species
*lasA*-*B*, *lasI*, *lasR *(quorum sensing genes)	Elastase	Elastase-*lasB*, autoinducer synthesis protein-*lasI*	Type II	Protease and elastolytic activity, staphylolytic activity, corneal damage, fibrinolytic, and collagenase, help in the tissue invasion, adaptation, and response to environmental stresses such as oxidative, heat, heavy metal, and salt stresses
lipI	Lipase	Lipase	Type II/Type VI	Phospholipase that hydrolyzes phosphatidic acid to produce lysophosphatidic acid
*motA*-*D*, *Y*	Motor proteins	Flagellar motor protein	Cytoplasmic membrane proteins	Protein transport, plasma membrane
*muc*-*E*, *P*	Mucin	Serine protease, a negative regulator for alginate biosynthesis	Type III	Zinc metalloprotease, proteolytic activity, hydrolase activity, catalytic activity, biofilm dispersal
*hisF2*, *H2*	Histidine	histidine transporter permease *hisQ*, histidine transporter *hisP*, phosphoribosyl-ATP pyrophosphatase-*hisE*, periplasmic histidine-binding protein *hisJ*, histidinol dehydrogenase-*hisD*, imidazole glycerol phosphate synthase subunit *hisF1*, imidazoleglycerol-phosphate dehydratase, histidinol-phosphate aminotransferase-*hisC2*	Type III	Aminoacid metabolic process, histidine biosynthetic process, hydrolase activity
*pvcA*-*D*	Pyoverdine chromophore	paerucumarin biosynthesis protein *pvcD*	Type VI	Influences biofilm development
*phzA1*-*A2*, *phzB1*-*G1*, *phzG2*,* phzH*, *phzM*, *phzS*	Pyocyanin	Phenazine biosynthesis protein	Type II (general secretion pathway’ and promotes outer membrane translocation of large (including some multimeric) exoproteins that are already folded in the periplasm)	Cytotoxic to cells, tissues, immune cells, cause apoptosis, release free radicals
plcH	Phospholipase	non-hemolytic phospholipase C-*plcC*, hemolytic phospholipase C	Type II	Disturbance of membrane lipid metabolism, tissue invasion
*pvdA*, *pvdD*-*J*, *pvdL*-*Q*, *pvdS*, *pvdY*	Pyoverdin	Pyoverdine biosynthetic protein, diaminobutyrate--2-oxoglutarate aminotransferase, protein *pvdJ*, protein pvdR, protein *pvdP*, pyoverdine synthetase F, extracytoplasmic-function sigma-70 factor, 3-oxo-C12-homoserine lactone acylase *pvdQ*, peptide synthase-*pvdL*, pyoverdine biosynthesis protein *pvdE*, L-ornithine N5-oxygenase-*pvdA*	Unknown	Carry iron and other metals that are required to form biofilms
*fimL*, *T*-*V*, *fimX*	Type 4a pili	hypothetical protein, type 4 fimbrial biogenesis protein FimU, protein FimX, Motility protein FimV, type 4 fimbrial biogenesis protein FimT	Unknown	Mannose-binding pili, cell adhesion, biofilm formation,
*fliA*, *C*-*S*	Flagellar protein	flagellar hook-basal body protein *fliE*, flagellin type B-*fliC*, flagellar protein *fliO*, flagellar biosynthesis protein *fliP*, flagellar biosynthesis chaperone-*fliJ*, flagellar biosynthesis sigma factor-*fliA*, flagellar-ring protein, flagellar motor switch protein G, flagellar motor switch protein-*fliN*, flagellar capping protein fliD, flagellum-specific ATP synthase-*fliI*	Type III/Type VI	RNA polymerase sigma factor for flagella operon, transcription regulatory activity, catalytic activity on RNA
*fpvI*, *fpvA*	Ferripyoverdine receptor	RNA polymerase sigma factor, ferripyoverdine receptor-*fpvA*, protein *fpvR*	Unknown	Siderophore uptake transmembrane protein
flhA	Flagellar biosynthesis protein	flagellar biosynthesis protein *flhA*	Type III	Flagellum-dependent motility, chemotaxis
*pchA*-*I*, *R*,	Pyochelin (Siderophore)	isochorismate-pyruvate lyase-*pchB*, pyochelin biosynthetic protein *pchC*, dihydroaeruginoic acid synthetase, salicylate biosynthesis isochorismate synthase, transcriptional regulator *pchR*, pyochelin biosynthetic protein PchG	Type II	Virulence
*pcr1*-*4*, *pcrD*, *pcrG*, *pcrH*, *pcrR*, *pcrV*	Translocator	Regulator in type III secretion, type III secretory apparatus protein *pcrD*, type III secretion protein *pcrV*, regulatory protein *pcrH*	Type III	Negative regulation of protein secretion
pldA	Phospholipase D	Phospholipase D	Unknown	Catalyzes hydrolysis of the phosphodiester bonds of glycerophospholipids—the main component of cell membranes—and assists the invasion
*flgA*-*N*	Flagella	flagellar basal body rod protein *flgG*, flagellar hook protein *flgE*, flagellar basal body L-ring protein, protein *flgM*, flagellar hook-associated protein *flgK*, flagellar hook-associated protein *flgL*, flagellar basal body P-ring biosynthesis protein *flgA*-*flgI*, flagellar basal body rod modification protein-*flgD*, flagellar basal body rod protein *flgF*, flagellar basal-body rod protein *flgB*	Unknown	Motility
*fliA*, *C*-*S*	Flagellar protein	Flagellar biosynthesis protein *fliQ*,	Type III	Flagellar assembly, swimming motility, chemotaxis, adhesion, and biofilm formation abilities
*pscB*-*L*, *pscN*-*U*	Chaperones	translocation protein in type III secretion, type III export protein *pscG*, type III secretion outer membrane protein *pscC*, type III secretion system protein, type III export protein *pscI*, type III export apparatus protein-*pscB*, type III export protein *pscJ *type III export protein *pscK*, type III export protein *pscF*	Type III (controls the injection of toxic proteins, called effectors, directly into the cytosol of host cells)	Delivers effector toxins directly from the bacteria into the host cytosol
*fleI*/*flag*, *fleN*, *fleP*/*fliT*, *fleQ*-*S*	Flagellar gene	two-component response regulator, flagellar synthesis regulator *fleN*, transcriptional regulator *fleQ*, two-component sensor-*fleS*	Unknown	Controls the transcriptional regulation of extracellular polysaccharide biosynthesis genes
stk1	Serine-threonine kinase	Catalyzing protein phosphorylation	Unknown	Cell wall metabolism
*pilA*-*C*, *pilE*-*K*, *pil*-*X*, *pilY1*-*Y2*, *pilZ*	Type 4 pili	two-component sensor *pilS*, type 4 fimbrial biogenesis protein *pilZ*, type 4 fimbrial biogenesis protein *pilY1*, type 4 fimbrial biogenesis protein *pilY2*, type 4 fimbrial biogenesis protein *pilX*, type 4 fimbrial biogenesis protein *pilF*, type 4 fimbrial biogenesis protein pilM, methyltransferase *pilK *type 4 fimbrial biogenesis protein *pilV*, type 4 fimbrial *pilA*, type 4 fimbrial biogenesis outer membrane protein *pilQ*, type 4 fimbrial biogenesis protein *pilB*, type 4 prepilin peptidase *pilD*, twitching motility protein *pilI*, type 4 fimbrial biogenesis protein *pilP*	Type IV	Biofilm formation
*popB*, *popD*, *popN*	Translocator	Translocator protein *popB*, type III secretion outer membrane protein *popN*, translocator outer membrane protein *popD*	Type III	Pore formation
*ptxS*, *ptxR*	*toxA *positive regulatory gene	Transcriptional regulator *ptxR*, *txpS*	Unknown	Regulate *exoA *secretion
*rhlA*-*C*, *rhlI*, *rhlR *(quorum sensing genes)	Rhamnolipids	*rhlR*-rhamnosyltransferase subunit B, rhamnosyltransferase 2 (*rhlC*), autoinducer synthesis protein *rhlI*, rhamnosyltransferase subunit A-*rhlA*	Unknown	Hemolytic, cytotoxic, harms respiratory epithelial cells, destroys ciliary functions, eliminates polymorphonuclear leucocytes, stabilizes biofilm, and helps motility, adaptation, and response to environmental stresses such as oxidative, heat, heavy metal, and salt stresses
*rpoN*, *rpoS*	RNA polymerase	RNA polymerase sigma factor *rpoS*	Unknown	Nitrogen-fixing biofilms
Stp1	Serine/threonine phosphoprotein	Serine/threonine phosphoprotein phosphatase *stp1*	Unknown	Cell wall synthesis and cell division
toxA	Exotoxin	Transcriptional regulator ToxR	Type II	Antiphagocytic, cytotoxic, inhibits protein synthesis, tissue damage, invasive property, keratitis, immunosuppression
pppA	PP2C-family protein phosphatase	A key regulatory component of the type VI secretory system	Type VI (survival advantage delivering toxins and translocating protein effectors into the host cells, acts as virulence factor, and takes part in biofilm formation)	Plays a key role in the disassembly and reassembly of type VI secretory system organelles
*xcpP*-*Z*	Extracellular protein deficient	Secretion pathway protein with less known functions, the pseudopilin *xcpT-X*, the putative ATPase *xcpR*, the polytopic inner membrane protein *xcpS*, the secretin *xcpQ*, and the peptidase *xcpA*	Type II	Assembly of Type IV pilus system
tag	DNA-3-methyladenine glycosylase activity	DNA-3-methyladeniine glycosylase activity	Type VI	DNA repair, catalytic activity
tse	Type six exported proteins	Type six exported proteins	Type VI	Toxin component of a toxin-immunity system and to arrest the growth of prokaryotic and eukaryotic cells
*tsr*	Chemotaxis transducer gene	Methyl-accepting chemotaxis protein I	Unknown	Respond to changes in the concentration of attractants and repellents in the environment, allow adaptation, cell motility
vfr	Virulence factor regulator (Vfr)	Key regulator of the type I-F CRISPR-Cas system in *P. aeruginosa*	Unknown	Regulates quorum sensing, pyocyanin, elastase, and exotoxin A production
vgr	Valine-glycine repeat (*vgr*) family proteins	Valine-glycine repeat (vgr) family proteins	Type VI	Similar to bacteriophage tube and tail spike proteins
*Wbp*/*waa*	Glycosyltransferase genes	Glycosyltransferase	Unknown	Core oligosaccharide biosynthesis
wzz	Lipopolysachharide O- antigen	O-antigen chain length regulator	Unknown	Lipopolysaccharide biosynthetic process

The strain-wise details of the sequence type based on MLST, virulence genes, and plasmid replicons are shown in Table 5.

**Table 5:** The strain-wise details of the virulence genes and MLST types PAE: *Pseudomonas aeruginosa;* PA: *Pseudomonas aeruginosa;* MLST: multilocus sequence typing; ST: sequence type

Strain	MLST type	Virulence genes
PAE-470	ST-1684	PA1458-1459, PA1464, PA1511, PA1656-1669, PA2359-2371, PA2373, PA2383-2384, PA3142-3143, PA3157, PA3348-3349, PA4218-4220, alg44, 8, algA-G, I-L, algP/algR3, Q-R, U, W-X, Z, aprA, cheY, Z, chp-E, clpV1, crc, dotU1, exlA, exoS, T, Y, exsA-E, fapA-F, fepD, fha1, fimL, T-V, fimX, fleI/flag, fleN, fleP/fliT, fleQ-S, flgA-N, flhA-B, flhF, fliA, C-S, fptA, fpvA, fpvI, fpvR, hcp1, hcpA, hisF2, hisH2, hsiA1,hsiB1/vipA/tssB,hsiC1/vipB/tssC, hsiE1, hsiF1/tssE, hsiG1/tssF, hsiH1/tssG, hsiJ1, icmF1/tssM1, lasA-B, lasI, lasR, lip1, mbtH-like, motA-D, Y, mprA, muc-E, P, pchA-I, R, pcr1-4, pcrD, pcrG, pcrH, pcrR, pcrV, phzA1-A2, phzB1-G1, phzG2, phzH, phzM, phzS, pilA-C, pilE-K, pil-X, pilY1-Y2, pilZ, plcH, popB, popD, popN, ppkA, pppA, pscB-L, pscN-U, ptxR, pvcA-D, pvdA, pvdF-J, pvdL-Q, pvdS, pvdY, rhlA-C, rhlI, rhlR, rpoN, rpoS, spcS, stk1, stp1, tagF/pppB, tagQ-T, toxA, tse1-3, tse7, tsr, vfr, vgrG1a-G1b, waaA, waaC, waaF-G, waaP, wbpA-B, wbpD-E, wbpG-M, wzx, wzy, wzz, xcpA/pilD, xcpP-Z
PAE-471	ST-244	PA1458-1459, PA1464, PA1511, PA1656-1669, PA2359-2371, PA2373, PA2383-2384, PA3142-3143, PA3157, PA3348-3349, PA4218-4220, alg44, 8, algA-G, I-L, algP/algR3, Q-R, U, W-X, Z, aprA, cheY, Z, chp-E, clpB, clpV1, crc, dotU1, exlA, exoS, T, Y, exsA-E, fapA-F, fepD, fha1, fimL, T-V, fimX, fleI/flag, fleN, fleP/fliT, fleQ-S, flgA-N, flhA-B, flhF, fliA, C-S, fptA, fpvA, fpvI, fpvR, hcp1, hcpA, hisF2, hisH2, hsiA1, hsiB1/vipA/tssB, hsiC1/vipB/tssC, hsiE1, hsiF1/tssE, hsiG1/tssF, hsiH1/tssG, hsiJ1, icmF1/tssM1, lasA-B, lasI, lasR, lip1, mbtH-like, motA-D, Y, mprA, muc-E, P, pchA-I, R, pcr1-4, pcrD, pcrG, pcrH, pcrR, pcrV, phzA1-A2, phzB1-G1, phzG2, phzH, phzM, phzS, pilA-C, pilE-K, pil-X, pilY1-Y2, pilZ, plcH, pldA, popB, popD, popN, ppkA, pppA, pscB-L, pscN-U, ptxR, pvcA-D, pvdA, pvdD-J, pvdL-Q, pvdS, pvdY, rhlA-C, rhlI, rhlR, rpoN, rpoS, spcS, stk1, stp1, tagF/pppB, tagQ-T, toxA, tse1-3, tse7, tsr, vfr, vgrG1a-G1b, waaA, waaC, waaF-G, waaP, wbpA-B, wbpD-E, wbpG-M, wzx, wzy, wzz, xcpA/pilD, xcpP-Z
PAE-472	ST-1029	PA1458-1459, PA1464, PA1511, PA1656-1669, PA2359-2371, PA2373, PA2383-2384, PA3348-3349, PA4218-4220, alg44, 8, algA-G, I-L, algP/algR3, Q-R, U, W-X, Z, aprA, cheY, Z, chp-E, clpB, clpV1, crc, dotU1, exlA, exoS, T, Y, exsA-E, fapA-F, fepD, fha1, fimL, T-V, fimX, fleI/flag, fleN, fleP/fliT, fleQ-S, flgA-N, flhA-B, flhF, fliA, C-S, fptA, fpvI, fpvR, hcp1, hcpA,hsiA1,hsiB1/vipA/tssB,hsiC1/vipB/tssC, hsiE1, hsiF1/tssE, hsiG1/tssF, hsiH1/tssG, hsiJ1, icmF1/tssM1, lasA-B, lasI, lasR, lip1, mbtH-like, motA-D, Y, mprA, muc-E, P, pchA-I, R, pcr1-4, pcrD, pcrG, pcrH, pcrR, pcrV, phzA1-A2, phzB1-G1, phzG2, phzH, phzM, phzS, pilA-C, pilE-K, pil-X, pilY1-Y2, pilZ, plcH, popB, popD, popN, ppkA, pppA, pscB-L, pscN-U, ptxR, pvcA-D, pvdA, pvdF-I, pvdL-Q, pvdS, pvdY, rhlA-C, rhlI, rhlR, rpoN, rpoS, spcS, stk1, stp1, tagF/pppB, tagQ-T, toxA, tse1-3, tse7, tsr, vfr, vgrG1a-G1b, waaA, waaC, waaF-G, waaP, wbpM, xcpA/pilD, xcpP-Z
PAE-473	ST-1978	PA1458-1459, PA1464, PA1511, PA1656-1669, PA2383-2384, PA3142-3143, PA3348-3349, PA4218, alg44, 8, algA-G, I-L, algP/algR3, Q-R, U, W-X, Z, aprA, cheY, Z, chp-E, clpV1, crc, dotU1, exlA-B, exoS, fapF, fha1, fimL, T-V, fimX, fleI/flag, fleN, fleP/fliT, fleQ-S, flgA-N, flhA-B, flhF, fliA, C-S, fptA, fpvA, fpvI, fpvR, hcp1, hcpA, hsiA1, hsiB1/vipA/tssB,hsiC1/vipB/tssC, hsiE1, hsiF1/tssE, hsiG1/tssF, hsiH1/tssG, hsiJ1, icmF1/tssM1, lasA-B, lasI, lasR, lip1, mbtH-like, motA-D, Y, mprA, muc-E, P, pchA-I, R, phzA1-A2, phzB1-G1, phzM, phzS, pilA-C, pilE-K, pil-X, pilY1-Y2, pilZ, ppkA, pppA, ptxR, pvcA-D, pvdA, pvdF-I, pvdL-Q, pvdS, rhlA-B, rhlI, rhlR, rpoN, rpoS, stk1, stp1, tagF/pppB, tagQ-T, tse1-3, tse7, tsr, vfr, vgrG1a-G1b, waaA, waaC, waaF-G, waaP, wbpM, xcpA/pilD, xcpP-Z
PAE-474	ST-485	PA1458-1459, PA1464, PA1511, PA1656-1669, PA2359-2371, PA2373, PA2383-2384, PA3142-3143, PA3348-3349, PA4218-4220, alg44, 8, algA-G, I-L, algP/algR3, Q-R, U, W-X, Z, aprA, cheY, Z, chp-E, clpV1, crc, dotU1, exoS, T, Y, exsA-E, fapA-F, fepD, fha1, fimL, T-V, fimX, fleN, fleQ-S, flgAK, M, N, flhA-B, flhF, fliA, C, E-R, fptA, fpvA, fpvI, fpvR, hcp1, hcpA, hsiA1,hsiB1/vipA/tssB,hsiC1/vipB/tssC, hsiE1, hsiF1/tssE, hsiG1/tssF, hsiH1/tssG, hsiJ1, icmF1/tssM1, lasA-B, lasI, lasR, lip1, mbtH-like, motA-D, Y, mprA, muc-E, P, pchA-I, R, pcr1-4, pcrD, pcrG, pcrH, pcrR, pcrV, phzA1-A2, phzB1-F1, phzG2, phzH, phzM, phzS, pilA-C, pilE-K, pil-X, pilY1-Y2, pilZ, plcH, popB, popD, popN, ppkA, pppA, pscB-L, pscN-U, ptxR, pvcA-D, pvdA, pvdD-J, pvdL-Q, pvdS, pvdY, rhlA-C, rhlI, rhlR, rpoN, rpoS, spcS, stk1, stp1, tagF/pppB, tagQ-T, toxA, tse1-3, tse7, tsr, vfr, vgrG1a-G1b, waaA, waaC, waaF-G, waaP, wbpM, xcpA/pilD, xcpP-Z
PAE-475	ST-2041	PA1458-1459, PA1464, PA1511, PA1656-1669, PA2359-2371, PA2373, PA2383-2384, PA3142-3143, PA3157, PA3348-3349, PA4218-4220, alg44, 8, algA-G, I-L, algP/algR3, Q-R, U, W-X, Z, aprA, cheY, Z, chp-E, clpV1, crc, dotU1, exlA, exoS, T, Y, exsA-E, fapA-F, fepD, fha1, fimL, T-V, fimX, fleI/flag, fleN, fleP/fliT, fleQ-S, flgA-N, flhA-B, flhF, fliA, C-S, fptA, fpvI, fpvR, hcp1, hcpA, hisF2,hisH2,hsiA1,hsiB1/vipA/tssB,hsiC1/vipB/tssC, hsiE1, hsiF1/tssE, hsiG1/tssF, hsiH1/tssG, hsiJ1, icmF1/tssM1, lasA-B, lasI, lasR, lip1, mbtH-like, motA-D, Y, mprA, muc-E, P, pchA-I, R, pcr1-4, pcrD, pcrG, pcrH, pcrR, pcrV, phzA1-A2, phzB1-G1, phzG2, phzH, phzM, phzS, pilA-C, pilE-K, pil-X, pilY1-Y2, pilZ, plcH, popB, popD, popN, ppkA, pppA, pscB-L, pscN-U, ptxR, pvcA-D, pvdA, pvdF-J, pvdL-Q, pvdS, pvdY, rhlA-C, rhlI, rhlR, rpoN, rpoS, spcS, stk1, stp1, tagF/pppB, tagQ-T, toxA, tse1-3, tse7, tsr, vfr, vgrG1a-G1b, waaA, waaC, waaF-G, waaP, wbpA-B, wbpD-E, wbpG-M, wzx,wzy, wzz, xcpA/pilD, xcpP-Z
PAE-476	ST-1974	PA1458-1459, PA1464, PA1511, PA1656-1669, PA2359-2371, PA2373, PA2383-2384, 3143, PA3348-3349, PA4218-4220, alg44, 8, algA-G, I-L, algP/algR3, Q-R, U, W-X, Z, aprA, cheY, Z, chp-E, clpB, clpV1, crc, dotU1, exlA, exoS, T, Y, exsA-E, fapA-F, fepD, fha1, fimL, T-V, fimX, fleI/flag, fleN, fleP/fliT, fleQ-S, flgA-N, flhA-B, flhF, fliA, C-S, fptA, fpvA, fpvI, fpvR, hcp1, hcpA, hsiA1, hsiB1/vipA/tssB, hsiC1/vipB/tssC, hsiE1, hsiF1/tssE, hsiG1/tssF, hsiH1/tssG, hsiJ1, icmF1/tssM1, lasA-B, lasI, lasR, lip1, mbtH-like, motA-D, Y, mprA, muc-E, P, pchA-I, R, pcr1-4, pcrD, pcrG, pcrH, pcrR, pcrV, phzA1-A2, phzB1-G1, phzG2, phzH, phzM, phzS, pilA-C, pilE-K, pil-X, pilY1-Y2, pilZ, plcH, popB, popD, popN, ppkA, pppA, pscB-L, pscN-U, pseB, ptxR, pvcA-D, pvdA, pvdD, F-I, pvdL-Q, pvdS, pvdY, rhlA-C, rhlI, rhlR, rpoN, rpoS, spcS, stk1, stp1, tagF/pppB, tagQ-T, toxA, tse1-3, tse7, tsr, vfr, vgrG1a-G1b, waaA, waaC, waaF-G, waaP, wbpM, xcpA/pilD, xcpP-Z
PAE-477	ST-646	PA1458-1459, PA1464, PA1511, PA1656-1669, PA2359-2371, PA2373, PA2383-2384, PA3142-3143, PA3157, PA3348-3349, PA4218-4220, alg44, 8, algA-G, I-L, algP/algR3, Q-R, U, W-X, Z, aprA, cheY, Z, chp-E, clpV1, crc, dotU1, exlA, exoS, T, Y, exsA-E, fapA-F, fepD, fha1, fimL, T-V, fimX, fleI/flag, fleN, fleP/fliT, fleQ-S, flgA-N, flhA-B, flhF, fliA, C-S, fptA, fpvI, fpvR, hcp1, hcpA, hsiA, hsiB1/vipA/tssB, hsiC1/vipB/tssC, hsiE1, hsiF1/tssE, hsiG1/tssF, hsiH1/tssG, hsiJ1, icmF1/tssM1, lasA-B, lasI, lasR, lip1, mbtH-like, motA-D, Y, mprA, muc-E, P, pchA-I, R, pcr1-4, pcrD, pcrG, pcrH, pcrR, pcrV, phzA1-A2, phzB1-G1, phzG2, phzH, phzM, phzS, pilA-C, pilE-K, pil-X, pilY1-Y2, pilZ, plcH, pldA, popB, popD, popN, ppkA, pppA, pscB-L, pscN-U, pseB, ptxR, pvcA-D, pvdA, pvdD-I, pvdL-Q, pvdS, rhlA-C, rhlI, rhlR, rpoN, rpoS, spcS, stk1, stp1, tagF/pppB, tagQ-T, toxA, tse1-3, tse7, tsr, vfr, vgrG1a-G1b, waaA, waaC, waaF-G, waaP, wbpM, xcpA/pilD, xcpP-Z
PAE-478	ST-646	PA1458-1459, PA1464, PA1511, PA1656-1669, PA2359-2371, PA2373, PA2383-2384, PA3142-3143, PA3348-3349, PA4218-4220, alg44, 8, algA-G, I-L, algP/algR3, Q-R, U, W-X, Z, aprA, cheY, Z, chp-E, clpV1, crc, dotU1, exlA, exoS, T, Y, exsA-E, fapA-F, fepD, fha1, fimL, T-V, fimX, fleI/flag, fleN, fleP/fliT, fleQ-S, flgA-N, flhA-B, flhF, fliA, C-S, fptA, fpvI, fpvR, hcp1, hcpA, hsiA1, hsiB1/vipA/tssB, hsiC1/vipB/tssC, hsiE1, hsiF1/tssE, hsiG1/tssF, hsiH1/tssG, hsiJ1, icmF1/tssM1, lasA-B, lasI, lasR, lip1, mbtH-like, motA-D, Y, mprA, muc-E, P, pchA-I, R, pcr1-4, pcrD, pcrG, pcrH, pcrR, pcrV, phzA1-A2, phzB1-F1, phzG2, phzH, phzM, phzS, pilA-C, pilE-K, pil-X, pilY1-Y2, pilZ, plcH, pldA, popB, popD, popN, ppkA, pppA, pscB-L, pscN-U, pseB, ptxR, pvcA-D, pvdA, pvdF-I, pvdL-Q, pvdS, rhlA-C, rhlI, rhlR, rpoN, rpoS, spcS, stk1, stp1, tagF/pppB, tagQ-T, toxA, tse1-3, tse7, tsr, vfr, vgrG1a-G1b, waaA, waaC, waaF-G, waaP, wbpM, xcpA/pilD, xcpP-Z

Functions of the plasmid replicons and the housekeeping genes based on which the MLST typing was performed are detailed in Table 6.

**Table 6:** Description of the functions of housekeeping genes and plasmid replicons DNA: deoxyribonucleic acid; GMP: guanosine monophosphate; XMP: xanthosine monophosphate; NADH: nicotinamide adenine dinucleotide hydrogen; NDH: NADH dehydrogenase-like complex; FMN: flavin mononucleotide

Virulence/housekeeping gene	Function	Usefulness
acsA	Acetyl-CoA synthetase catalyzes the conversion of acetate into acetyl-CoA (AcCoA), necessary for anabolic and catabolic pathways	Helps in growth and sporulation
aroE	Shikimate dehydrogenase (NADP(+)): biosynthesis of the chorismite and aromatic amino acids	Microbial cell survival
guaA	GMP Synthase: catalyzes the synthesis of GMP from XMP	Survival, colonization, and infectivity of bacteria in the host
mutL	DNA mismatch repair protein: repairs mismatches in DNA	Hypermutability of strains
nuoD	NADH-quinone oxidoreductase subunit D: NDH-1 shuttles electrons from NADH, via FMN and iron-sulfur (Fe-S) centers, to quinones in the respiratory chain	Conserves the redox energy in a proton gradient
ppsA	Phosphoenolpyruvate synthase: catalyzes the phosphorylation of pyruvate to phosphoenolpyruvate	Bacterial growth, gluconeogenesis and virulence
trpE	Anthranilate synthase component 1: catalyzes the two-step biosynthesis of anthranilate, an intermediate in the biosynthesis of L-tryptophan	Bacterial survival in the macrophages and evade host responses
*IncFIA*, *IncFIB*, *IncFII *(*pSE11*)	Incompatibility (Inc) group	Carry drug-resistance genes
ColKP3	Col-like plasmid replicons	Carry drug-resistance genes

## Discussion

The molecular characterization of PA clinical isolates through WGS provides critical insights into the genetic underpinnings of antibiotic resistance and virulence. Our study identified a concerning prevalence of antibiotic-resistant genes among the nine PA isolates analyzed. All isolates harbored multiple resistance genes, including *bla*_PDC_ and *bla*_OXA, _and were associated with beta-lactam resistance. The presence of *bla*_VIM_, a metallo-beta-lactamase, in one isolate underscores the potential for severe treatment challenges due to resistance to carbapenems, a last resort antibiotic class.

Identifying various virulence genes, such as *toxA*, *fih*, and *pvd*, is critical for understanding the pathogenic potential of PA. These genes are implicated in toxin production and biofilm formation, which enhance the bacterium's ability to establish infections and evade host defenses. The presence of these virulence determinants in all isolates indicates a uniformly high risk of pathogenicity among the strains studied. This finding emphasizes the importance of monitoring virulence gene profiles in clinical settings, as strains with a robust virulence arsenal are more likely to cause severe infections, particularly in immunocompromised patients.

Antibiotic resistance genes

The pseudomonas-derived cephalosporinase (*bla*_PDC_) was the most frequent AMR gene. This gene was identified in all the isolates included in this study. The AMR gene *bla*_PDC_ is chromosomally derived and confers high-level cephalosporin resistance through mutations and catalytic activity. A recent study revealed PA isolates demonstrating *bla*_PDC_ showing resistance to newly introduced cephalosporins like ceftolozane, despite this antibiotic not being prescribed to the patient. This confirms the potential for cross-reactive AMR among the PA isolates with *bla*_PDC_ even in the absence of selective pressure [20].

The ecological and evolutionary mechanisms involved in the development of AMR have not yet been completely elucidated. WGS is a potential tool that could enable a deeper understanding of the molecular mechanisms bacterial strains employ to develop AMR [21]. A previous study that analyzed the *bla*_PDC_ gene revealed that its ancestor could have evolved more than 4000 years ago. This demonstrates the highly conserved nature of *bla*_PDC_ wherein the bacterial strains with this gene confer similar antibiotic resistance patterns [22].

In recent times, there has been increased evidence of the prevalence and spread of multidrug-resistant (MDR), extensively drug-resistant (XDR), and pan-drug-resistant (PDR) strains of PA and other bacterial species [12,23]. Oxacillinase (*bla*_OXA_) and Verona integron-encoded metallo-beta-lactamase (*bla*_VIM_) are the two carbapenemases detected in the strains included in this study. A very high percentage of PA strains showing resistance to the carbapenem group of antibiotics resulting in poor patient outcomes was noticed in countries like China (54%), South and Central America (69%), the Middle East, Australia (57%), and Singapore (57%). However, the USA revealed a low (2%) prevalence of carbapenem resistance [24].

The multi-locus sequence typing

This study showed the presence of ST244, a high-risk PA strain with global significance [25,26]. ST244 was not identified in a previous study from the United Kingdom. Other high-risk PA strains noticed in the UK but not identified in the present study were STs 111, 233, 235, 357, 654, and 773 [27]. Our study did not find the presence of *bla*_SHV_, *bla*_TEM_, and *bla*_NDM_ as evidenced by a study reported from Kenya [28].

A recent study from South Africa identified a *bla*_NDM-1_ carrying high-risk ST773 that is coded on the integrative and conjugative element (ICE) region and can potentially transmit among bacterial species. This study points to the significance of identifying the prevalence of high-risk strains and initiation of control and preventive measures [29].

The contradicting antibiotic susceptibility patterns noticed among the PA isolates in this study point to the fact that clinical decisions cannot be made solely based on phenotypic or genotypic susceptibility results. This was confirmed by a recent study that assessed anti-tubercular drug susceptibility patterns among *Mycobacterium tuberculosis *isolates [30].

Comparison of genotype and phenotypic antimicrobial susceptibility patterns

In the present study, we noticed discordant genotypic and phenotypic antibiotic susceptibility patterns concerning certain antibiotics, including co-trimoxazole (66.66% resistance phenotype vs. 100% sensitive genotype), aminoglycosides (100% sensitive phenotype vs. 100% resistant genotype), and beta-lactamase/extended-spectrum beta-lactamase (ESBL) (44.44% sensitive phenotype vs. 100% resistant genotype).

Understanding the antibiotic susceptibility patterns and interpretation of the genotypic and phenotypic drug susceptibility patterns of a bacteria isolated from the human clinical specimen is complex. If the genotypic results comply with the phenotypic results, the patient may be appropriately treated with the drugs. However, if the AMR gene is detected and the phenotype results demonstrate susceptibility to the drug, and in cases where the AMR gene is not detected and the phenotypic results show drug resistance, choosing an antibiotic to treat the patient is difficult [31]. However, the CLSI guidelines suggest the repetition of AST and checking for the purity of cultures, among others, to facilitate reporting such discrepancies [13].

Study limitations

This study included fewer PA isolates and did not compare the presence of AMR and virulence genes with the clinical characteristics and patient outcomes. Although all PA isolates were collected from a single institution, phylogenetic analysis to establish the relatedness of the strains included in this study was not conducted.

## Conclusions

The study results revealed discordant phenotypic and genotypic antibiotic susceptibility patterns. Most study strains demonstrated the presence of multiple AMR and virulence genes. This study showed the presence of ST244, a high-risk PA strain with global significance. PA is an opportunistic pathogen, and its isolation among hospitalized patients should be carefully evaluated. Tracking PA for the presence of high-risk STs and resistance and virulence genes could improve the understanding of the organism. Molecular data obtained from this study could potentially enable physicians to predict invasive infections and treatment failures. The data obtained from this study could be applied to devise treatment and management strategies favorable to patients and the hospital or healthcare institution.
